# Down regulation of SPAG9 reduces growth and invasive potential of triple-negative breast cancer cells: possible implications in targeted therapy

**DOI:** 10.1186/1756-9966-32-69

**Published:** 2013-09-19

**Authors:** Abhilasha Sinha, Sumit Agarwal, Deepak Parashar, Archana Verma, Shikha Saini, Nirmala Jagadish, Abdul S Ansari, Nirmal K Lohiya, Anil Suri

**Affiliations:** 1 Cancer Microarray, Genes and Proteins Laboratory, National Institute of Immunology, Aruna Asaf Ali Marg, New Delhi 110 067, India; 2 Centre for Advanced Studies, Department of Zoology, University of Rajasthan, Jaipur 302 004, India

**Keywords:** Migration, Invasion, CT antigens, SPAG9, Gene silencing

## Abstract

**Background:**

Recently, we reported an association of a novel cancer testis (CT) antigen, sperm-associated antigen 9 (SPAG9) expression in breast cancer clinical samples, indicating its potential role in carcinogenesis. Around 15% breast cancers are designated as triple-negative for which treatment modalities are limited. Therefore, in the present study, we assessed the role of SPAG9 in triple-negative breast cancer cells.

**Methods:**

SPAG9 mRNA and protein expression was investigated in various breast cancer cells of different hormone receptor status and different subtypes by employing reverse transcriptase-polymerase chain reaction (RT-PCR), real time PCR, Western blotting, indirect immunofluorescence (IIF) and fluorescence activated cell sorting (FACS). Employing plasmid-based small interfering RNA (siRNA) approach, knockdown of *SPAG9* was carried out in triple-negative breast cancer cells, MDA-MB-231, to assess its role on various malignant properties *in vitro* and *in vivo*.

**Results:**

*SPAG9* mRNA and protein expression was detected in all breast cancer cells. Further, IIF results showed that SPAG9 was predominantly localized in the cytoplasm of breast cancer cells. FACS analysis revealed distinct SPAG9 surface localization in breast cancer cells. Gene silencing of *SPAG9* resulted in significant reduction in cellular proliferation, colony forming ability, migration, invasion and cellular motility of MDA-MB-231 cells. Further, ablation of SPAG9 expression resulted in reduction in the tumor growth of human breast cancer xenograft in nude mice *in vivo*.

**Conclusions:**

In summary, our data indicated that down regulation of SPAG9 reduces growth and invasive potential of triple-negative breast cancer cells, suggesting that SPAG9 may be a potential target for therapeutic use.

## Background

Breast cancer is the most common cause of cancer-related deaths among women worldwide, with the highest mortality incidence in developing countries [1]. Breast cancer is a complex disease which has different histotypes and molecular subtypes based on molecular profiling with different prognostic and therapeutic implications. Recent studies have documented that breast cancer disease is a resultant of accumulation of genomic [2] and epigenomic [3] alterations resulting in reduced apoptosis, unchecked proliferation, increased motility and invasion abilities and metastasis in various other distant sites [4]. In this regard, understanding the underlying mechanisms involved in such process would eventually reveal the novel target molecules involved in the disease progression and may help in cancer treatment.

In clinical practice, breast cancer treatment modalities are based on the specific proteins that are expressed in cancerous tissue specimen. Majority of the breast cancer patients express proteins such as estrogen receptor (ER) and progesterone receptor (PR) for which targeted hormone therapy is available with better clinical outcome [5]. In addition, around 15-20% patients express human epidermal growth factor receptor 2 (HER2) protein, for which effective trastuzumab therapy is available with good prognosis [6]. In contrast, around 15% of diagnosed breast cancers are designated as triple-negative and are characterized as ER negative (ER^-^), PR negative (PR^-^) and HER2 negative (HER2^-^) [7]. Triple-negative breast cancer patients represent an important clinical challenge because these patients do not respond to endocrine therapy or any other available targeted agents. Therefore, it is necessary to investigate and characterize target molecules in triple-negative breast cancers for better cancer management.

Earlier few studies have reported the expression of novel proteins in triple-negative breast cancers; however none of these proteins have been used in clinical setup [8]. Therefore, it is important to characterize the novel targets to unravel the biological pathways and modes of progression in order to develop new candidate molecules and therapies. In this regard, a unique class of tumor antigens designated as cancer testis (CT) antigens has been reported to have aberrant expression in various tumors, restricted expression in the testis and no or low expression in other somatic tissues [9]. CT antigens have been proposed to play pivotal role in various malignant properties of cancer cells [10]. Employing gene silencing approach, knockdown of CT antigens could be specifically targeted and their involvement in carcinogenesis could be investigated which may lead to novel treatment modalities. Therefore, understanding the involvement of CT antigen may be a good approach to study the molecular mechanism involved in breast cancer.

Recently, we reported an association of sperm-associated antigen 9 (SPAG9) expression, a new member of CT antigen family, in various types of cancers [9]. Using plasmid-based small interfering RNA (siRNA) approach to knockdown *SPAG9*, we demonstrated significant reduction in cellular proliferation, colony forming ability, cellular migration, invasion and wound healing capacity in different types of cancers [11-13]. Interestingly, we also demonstrated an association of SPAG9 immuno-reactivity score (IRS) in early grades of breast cancer patients. In addition, 88% breast cancer specimens showed SPAG9 expression independent of tumor stages and grades [14]. Collectively, our data suggested that SPAG9 could be playing a potential role in various malignant properties of breast tumorigenesis.

In the present study, we investigated the SPAG9 expression in different breast cancer cell line models of different hormone receptor status and different subtypes. Further, involvement of SPAG9 was investigated for various malignant properties in triple-negative MDA-MB-231 cells, employing siRNA approach. Our data revealed that SPAG9 mRNA and protein expression was detected in all breast cancer cells. In addition, relative qPCR data demonstrated 20 to 52 folds higher expression of *SPAG9* mRNA in MCF-7, MDA-MB-231, BT-474 and SK-BR-3 breast cancer cells as compared to normal mammary epithelial cells. SPAG9 was also shown to be anchored on the plasma membrane of breast cancer cells. Employing gene silencing approach, knockdown of *SPAG9* gene revealed that SPAG9 plays an important role in cellular proliferation, colony forming ability, migration and invasion. Furthermore, *in vivo* breast xenograft studies in nude mice revealed that SPAG9 siRNA plasmid injected mice showed significant reduction in tumor growth. Collectively, our data has laid foundation for SPAG9 to be used as a potential therapeutic target for triple-negative breast cancer.

## Material and method

### Breast cancer cell lines

Four breast cancer cell lines of various subtypes, harboring different hormone receptors, such as MCF-7 (luminal-A, ER^+^ PR^+^ Her2^-^), BT-474 (luminal-B, ER^+^ PR^+^ Her2^+^), SK-BR-3 (HER2 overexpressing, ER^-^ PR^-^ Her2^+^) and MDA-MB-231 (highly metastatic basal, triple-negative ER^-^ PR^-^ Her2^-^) were used in the study and were procured from American Type Culture Collection (ATCC, Manassas, VA). All the cells were cultured in recommended medium under standard conditions. Human normal mammary epithelial cells were purchased and maintained according to manufacturer’s directions (Gibco, Life Technologies Corporation, Carlbad, CA).

### RNA isolation, reverse transcriptase-polymerase chain reaction (RT-PCR) and real-time PCR

*SPAG9* mRNA was detected in normal mammary epithelial cells and all breast cancer cells by extracting total RNA using RNeasy Mini kit (Qiagen GmbH, Hilden, Germany) and complementary DNA (cDNA) was synthesized using High-Capacity cDNA Reverse Transcription Kit (Applied Biosystems, Carlsbad, CA) by following the manufacturer’s protocol. RT-PCR was performed using cDNA template and *SPAG9* specific primers. Following *SPAG9* primers were designed from overlapping exons of *SPAG9* in order to avoid genomic DNA contamination during amplification: *SPAG9* Forward: 5′ *GGGG GAATTCGATCAGGAACTTAAGGAACAGCAGAAGGAG* 3′ *SPAG9* Reverse: 5′ *GGGG GGTACCCTGTTTCTCGTGCACCTGGCACACTTGCAA* 3′. RT-PCR was carried out by 30 amplification cycles- 1 cycle of denaturation at 94°C for 2 min, 30 cycles: denaturation at 94°C for 45 s; annealing at 50°C for 45 s; extension at 72°C for 2 min; and a final elongation cycle at 72°C for 7 min. Amplicon of samples were electrophoresed on 0.7% agarose gel and stained with ethidium bromide and photographed under UV light in EC3 Imaging System (UVP, Upland, CA). Further, *SPAG9* sequence was confirmed by cloning PCR product in TOPO vector (Invitrogen, Carlsbad, CA). *β-Actin* mRNA expression was used as an internal control. *SPAG9* mRNA expression was also checked in normal mammary epithelial cells as a negative control. Real-time PCR was done using 10 ng of cDNA from normal mammary epithelial cells and breast cancer cell lines mentioned above with SYBR Green Real time PCR master mix (Bio-Rad, CA, USA) on an iCycler iQ multicolour real time PCR detection system (Bio-Rad, CA, USA) according to manufacturer’s instructions*. β-Actin* was used as an internal control in all the reactions. *SPAG9* gene expression levels in each breast cancer cell line sample were subsequently normalized using expression level of *β-actin* in the same mRNA sample as a house keeping gene. All samples were measured in triplicates. Primers were as follows:

*SPAG9* Forward primer *5*′- *GAATTCGATCAGGAACTTAAGGAACAGCAGAAGGAG*-3′

*SPAG9* Reverse primer 5′-*GGTACCCTGTTTCTCGTGCACCTGGCACACTTGCAA-*3′

*β-actin* Forward primer *5′- ATCTGGCACCACACCTTCTACAATGAGCTGCG*-3′

*β-actin* Reverse primer *5′- CGTCATACTCCTGCTTGCTGATCCACATCTGC-3′*

### Western blotting

Endogenous SPAG9 protein expression was validated in all normal mammary epithelial cells and breast cancer cells by Western blot analysis. Cell lysates were prepared in lysis buffer [(1.5 mM Tris–HCl, pH 7.5, 150 mM NaCl, 0.5% sodium deoxycholate and 1% Nonidet P-40 (NP-40) plus 1X Protease inhibitor cocktail (Sigma-Aldrich, St. Louis, MO)]. The protein concentration of the cell lysates was determined by the bicinchoninic acid (BCA) method as described in the manufacturer’s protocol (Thermo Fisher Scientific Inc., Rockford, IL). Cell lysates (20 μg) were denatured in laemmli loading buffer [10% glycerol, 5% 2-mercaptoethanol, 2% sodium dodecyl sulphate, 62.5 mM Tris (pH 6.8), 0.05% bromophenol blue] and were resolved on 10% sodium dodecyl sulphate-polyacrylamide gel electrophoresis (SDS-PAGE) gel. Further, protein was electro-transferred to polyvinylidene difluoride (PVDF) membrane in order to detect SPAG9 protein expression. PVDF membranes were blocked with 5% non fat dry milk and were incubated with anti-SPAG9 polyclonal antibody (1:100 dilution) for 1h at RT. Subsequently, blots were incubated with horseradish peroxidase (HRP)-conjugated goat anti-rat IgG as a secondary antibody (Jackson ImmunoResearch Laboratories Inc., West Grove, PA) for 1h at RT. The blots were developed with the immobilon western chemiluminescent HRP substrate (Millipore Corporation, Billerica, MA) according to the manufacturer’s protocol. β-Actin was used as an intrinsic loading control for all cell lysates analyzed.

### Indirect immunofluorescence and fluorescence activated cell sorting

Indirect immunofluorescence (IIF) assays and fluorescence activated cell sorting (FACS) analysis were carried out to detect SPAG9 protein expression in breast cancer cells as described earlier [13]. For IIF assays, briefly, cells were fixed, permeabilized and were probed with anti-SPAG9 antibody, followed by fluorescein isothiocyanate (FITC)-conjugated goat anti-rat IgG as secondary antibody (Jackson ImmunoResearch Laboratories Inc., West Grove, PA). Cell nucleus was stained with 4’, 6-diamidino-2-phenylindole [(DAPI) Sigma-Aldrich, St. Louis, MO]. Subsequently, images were captured using confocal microscope [ZEISS LSM 510 Meta (Zeiss, Oberkochen, Germany)]. For FACS analysis, cells were harvested and analyzed for SPAG9 surface localization as described earlier [13]. Fixed cells were probed with anti-SPAG9 polyclonal antibody followed by goat anti-rat IgG conjugated with FITC as a secondary antibody. Cells stained with secondary antibody only were used to account for the background fluorescence. Data acquisition and analysis was done using CellQuest v3.3 software.

### Down regulation of *SPAG9* using small interfering RNA approach

In order to study the role of SPAG9 in various malignant properties of breast cancer cells, transient transfection was carried out in MDA-MB-231 cells using Lipofectamine (Invitrogen, Carlsbad, CA) reagent, as described previously [13]. Briefly, 6 μg of *SPAG9* specific siRNA (SPAG9 siRNA) and control siRNA (scrambled SPAG9) were used for the *in vitro* experiments. Cells were harvested 48 h post-transfection and cell lysate was prepared and analyzed by Western blotting as explained above.

### Cellular proliferation and colony formation assay

Cellular growth and colony forming ability were investigated in MDA-MB-231 cells post-transfection with plasmid driven siRNA as described previously [13]. To study the cellular proliferation, 2 × 10^4^ MDA-MB-231 cells transfected with 6 μg of SPAG9 siRNA or control siRNA were seeded in triplicates in 6-well plate. Cell number was counted with hemocytometer at three different time points after seeding for 24 h, 48 h and 72 h. For colony formation assay, a total of 400 to 1200 transfected cells were seeded into 6-well plates. Ten days post-seeding, the cells were fixed with 5% glutaraldehyde in Phosphate buffered saline (PBS) and stained with 0.5% toluidine blue (Sigma-Aldrich, St. Louis, MO). The colonies were manually counted after washing cells with PBS. Images of representative fields were captured using Nikon Eclipse E 400 microscope (Nikon, Fukok, Japan). Each experiment was repeated in triplicates.

### Migration and invasion assays

To study the involvement of SPAG9 in various malignant properties of breast cancer cells, cell migration and invasion assays were performed using BD Biosciences Boyden chamber (Becton Dickinson Labware, Bedford, MA), as described previously [13]. In migration assay, 2 × 10^5^ transfected cells in 500 μl of serum free medium were layered on the 8-μm pore inserts of the transwell membrane in triplicate wells of 24-well plate. Foetal bovine serum [(FBS) Biological Industries Israel Beit-Haemek Ltd. Kibbutz Beit-Haemek, Israel] supplemented (750 μl) medium was used as chemoattractant in the lower chamber. Cells thus migrated to the lower chamber of the wells were fixed with 5% glutaraldehyde in PBS, stained with 0.5% toluidine blue and were counted using bright field microscopy. For invasion assay, 8-μm pore inserts were coated with 15 μg of Matrigel as a basement barrier (Becton Dickinson Labware, Bedford, MA) and then 2 × 10^5^ transfected cells were layered. Cells that invaded through the artificial extracellular matrix and migrated to the lower compartment of the Boyden chamber were fixed and stained as explained above. Representative fields were photographed under Nikon Eclipse E 400 microscope (Nikon, Fukok, Japan). All the experiments were done in triplicates.

### Wound healing assay

Cellular motility was also studied by carrying out wound healing assay as described previously [13]. Cells transfected with 6 μg of SPAG9 siRNA or control siRNA were seeded at a density of 1 × 10^6^ on a 35-mm Petri dish. After overnight incubation, on the confluent cell monolayer, an artificial wound was carefully created using 200-μl filtered tip. Subsequently, the petri dishes were washed with serum free medium and cultured with 2% FBS medium and photomicrograph was taken immediately at 0 h. Photomicrographs were also taken at 12 h, 24 h and 48 h under Nikon Eclipse E 400 microscope (Nikon, Fukok, Japan). Within each assay the experiments were performed in triplicates.

### Breast cancer cells xenograft studies

To carry out *in vivo* studies, athymic nude mice (National Institute of Immunology [NII], National Institutes of Health, [S] nu/nu) were used in this study, after obtaining approval from animal ethical committee of National Institute of Immunology. Human tumor xenograft of breast MDA-MB-231 cells was established by injecting 5 ×10^6^ cells subcutaneously on the lower back, suspended in Matrigel collagen basement membrane (BD Biosciences, Bedford, MA). These nude mice were maintained at NII animal facility in a pathogen-free atmosphere. After two weeks, nude mice that had developed subcutaneous xenograft tumors of around 50–100 mm^3^ were divided into two treatment groups (8 mice in each group): SPAG9 siRNA and control siRNA. Control siRNA or SPAG9 siRNA plasmids (50 μg) suspended in 200 μl of PBS were injected intra-tumorally followed by a booster injection of 25 μg plasmid injected twice weekly for 7 weeks. Tumor growth was measured regularly twice a week. Tumor volume (V) was calculated by measuring tumor dimensions using digital calipers as described earlier [12]. At the end of the experiment, tumors were excised, fixed, embedded in paraffin and sectioned for histological examination of SPAG9 and PCNA expression.

### Immunohistochemical analysis

Immunohistochemical analysis was performed on 4-μm-thick sections of tumor tissue excised from control siRNA and SPAG9 siRNA mice using polyclonal anti-SPAG9 antibody and mouse anti-PCNA antibody as described earlier [11,12]. Briefly, sections were deparaffinized, rehydrated, washed with PBS (pH7.2) and were incubated in methanolic H_2_O_2_ (45:5) for 45 minutes to block and remove all traces of endogenous peroxidase. Subsequently, tissue sections were blocked with 5% normal goat serum for 1 hour at RT and probed with polyclonal anti-SPAG9 antibody for overnight at 4°C. After three washes with PBS, sections were incubated with Horse reddish peroxidase–conjugated goat anti-rat IgG (Jackson ImmunoResearch Laboratories, West Grove, PA) as a secondary antibody. After incubation sections were subjected to three washings with PBS and the color was developed using 3, 3′-Diaminobenzidine (Sigma- Aldrich, St. Louis, MO) as a substrate. Serial sections of same tissue specimens were also processed for immunohistochemical staining for PCNA using the same protocol. Slides were counterstained with hematoxylin solution, mounted and observed under a Nikon Eclipse E 400 microscope (Nikon, Fukuoka, Japan). Six random fields of each tissue section were examined by counting >500 cells under ×400 magnification.

### Statistical analysis

The statistical significance of the results of *in vitro* and *in vivo* data was determined by the Student’s *t* test using the SPSS version 20.0 statistical software package (SPSS Inc., Chicago, IL). A *P*-value of less than 0.05 was considered statistically significant. All experimental data are presented as mean ± standard error.

## Results

### *SPAG9* mRNA expression in breast cancer cell lines

**Figure 1:**
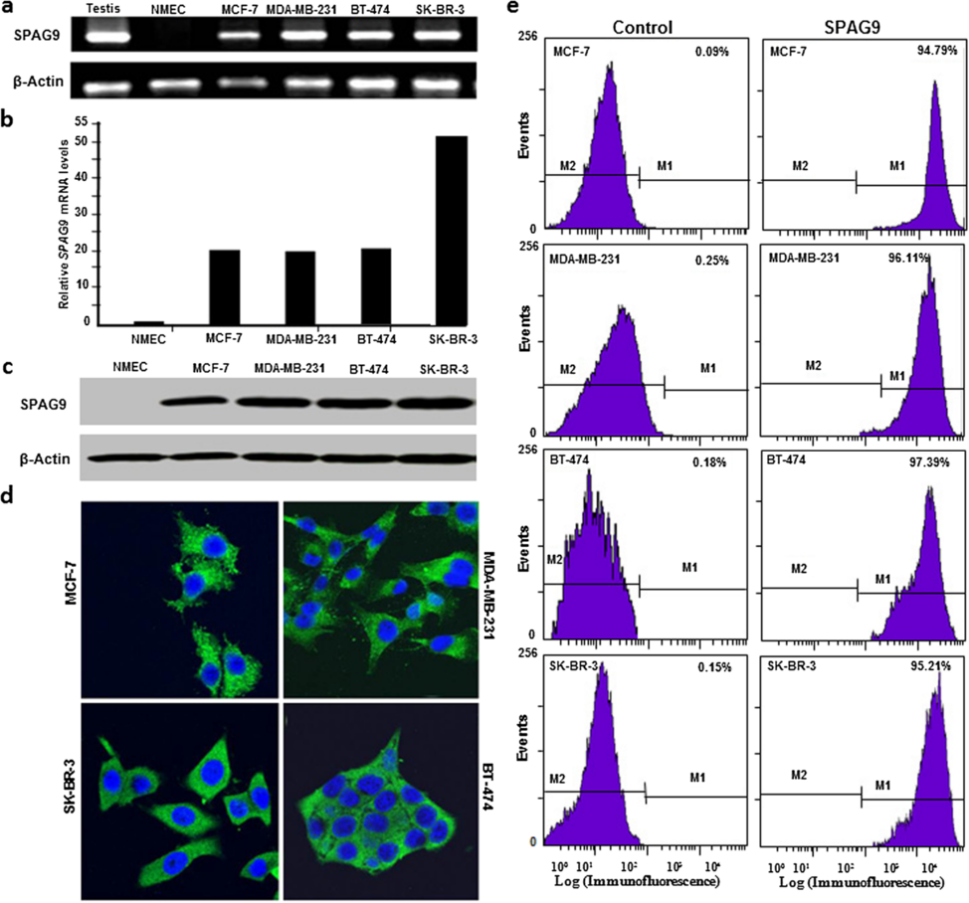
**SPAG9 expression in breast cancer cells. (a)** RT-PCR analysis showed *SPAG9* mRNA expression in testis and no expression in normal mammary epithelial cells (NMEC). *SPAG9* mRNA expression was observed in MCF-7, MDA-MB-231, BT-474 and SK-BR-3 cells. *β-Actin* gene expression was used as an internal control. **(b)** Relative expression of *SPAG9* mRNA in MCF7, MDA-MB-231, BT-474 and SK-BR-3 breast cancer cells relative to NMEC. **(c)** Validation of SPAG9 protein expression in NMEC and breast cancer cells by Western blot analysis. SPAG9 reactive band was detected in MCF-7, MDA-MB-231, BT-474 and SK-BR-3 cell lysates. However, no reactivity against SPAG9 was detected in NMEC. Lower panel depicts the β-actin protein reactivity as an internal loading control in all breast cancer cells. **(d)** SPAG9 protein expression in breast cancer cells by IIF assay. IIF assay revealed distinct cytoplasmic SPAG9 localization in fixed and permeabilized cells probed with anti-SPAG9 antibody in MCF-7, MDA-MB-231, BT-474 and SK-BR-3 cells. Nuclei of the cells were stained blue with DAPI. All images were captured using confocal microscope (Original magnification, ×630; objective, 63×). **(e)** SPAG9 surface localization in breast cancer cells. FACS analysis distinctly showed SPAG9 surface localization in MCF-7, MDA-MB-231, BT-474 and SK-BR-3 cells probed with anti-SPAG9 antibody as depicted in histogram plot showing displacement of fluorescence intensity on X axis (M1) as compared to fluorescence intensity of cells stained with secondary antibody only (M2). Representative plots showed high percentages of distinct population of MCF-7 (94.79%), MDA-MB-231 (96.11%), BT-474 (97.39%) and SK-BR-3 (95.21%) cells showing SPAG9 surface localization as compared to cells stained with secondary antibody only.

RT-PCR analysis revealed that *SPAG9* mRNA was found in all breast cancer cell line models used in the present study [MCF-7 (ER^+^/PR^+^/Her2^-^ luminal-A subtype), SK-BR-3 (ER^-^/PR^-^/Her2^+^ ERBB2 associated subtype), BT-474 (ER^+^/PR^+^/Her2^+^ triple-positive luminal-B subtype) and MDA-MB-231 (ER^-^/PR^-^/Her2^-^ triple-negative basal subtype)] as shown in Figure 1a. Human testis cDNA was used as positive control which also revealed same size PCR amplicon. Moreover, no expression of *SPAG9* transcript was detected in normal mammary epithelial cells which clearly indicated that *SPAG9* is expressed exclusively in cancerous cells. Further, PCR amplicon was subcloned in TOPO vector and sequenced. Nucleotide sequencing confirmed the *SPAG9* mRNA sequence in all breast cancer cells. Thus, our RT-PCR results indicated that *SPAG9* gene is expressed in all breast cancer cells independent of their hormone receptor status or subtypes. We further assessed *SPAG9* mRNA expression in normal mammary epithelial cells, MCF7, MDA-MB-231, BT-474 and SK-BR-3 breast cancer cell lines by quantitative real-time PCR. All breast cancer cell lines evaluated displayed higher levels of *SPAG9* expression, compared to control normal mammary cells (Figure 1b). *SPAG9* expression was around 20 fold higher in MCF7, MDA-MB-231 and BT-474. However, 52 fold higher *SPAG9* expression was observed in SK-BR-3 as compared to normal mammary cells.

### SPAG9 protein expression in breast cancer cell lines

To validate the *SPAG9* gene expression, endogenous SPAG9 protein expression was further investigated by Western blot analysis which revealed an immunoreactive band in all the four breast cancer cells as shown in Figure 1c. β-Actin reactive band revealed equal loading of the lysate protein prepared from all breast cancer cells. Subsequently, SPAG9 protein expression was investigated by IIF in fixed and permeabilized MCF-7, MDA-MB-231, BT- 474 and SK-BR-3 cells (Figure 1d), which revealed SPAG9 protein expression predominantly in cytoplasm. Further, surface localization of SPAG9 protein was detected in all four breast cancer cells as demonstrated by FACS analysis (Figure 1e). FACS analysis clearly showed the displacement of fluorescence intensity on the X-axis in breast cancer cells probed with anti-SPAG9 polyclonal antibody indicating SPAG9 surface localization in MCF-7, MDA-MB-231, BT-474 and SK-BR-3 cells (Figure 1e). FACS analysis also demonstrated high percentage of SPAG9 expressing cells showing SPAG9 surface localization in MCF-7 (94.79%), MDA-MB-231 (96.11%), BT-474 (97.39%) and SK-BR-3 (95.21%) cells. As expected, no or very low shift in fluorescence intensity was observed in cells probed with only secondary antibody. Collectively, IIF and FACS data suggested that SPAG9 may be a potential target for cancer immunotherapeutics.

### Gene silencing of *SPAG9* inhibits cellular proliferation and colony forming ability of MDA-MB-231 cells

**Figure 2:**
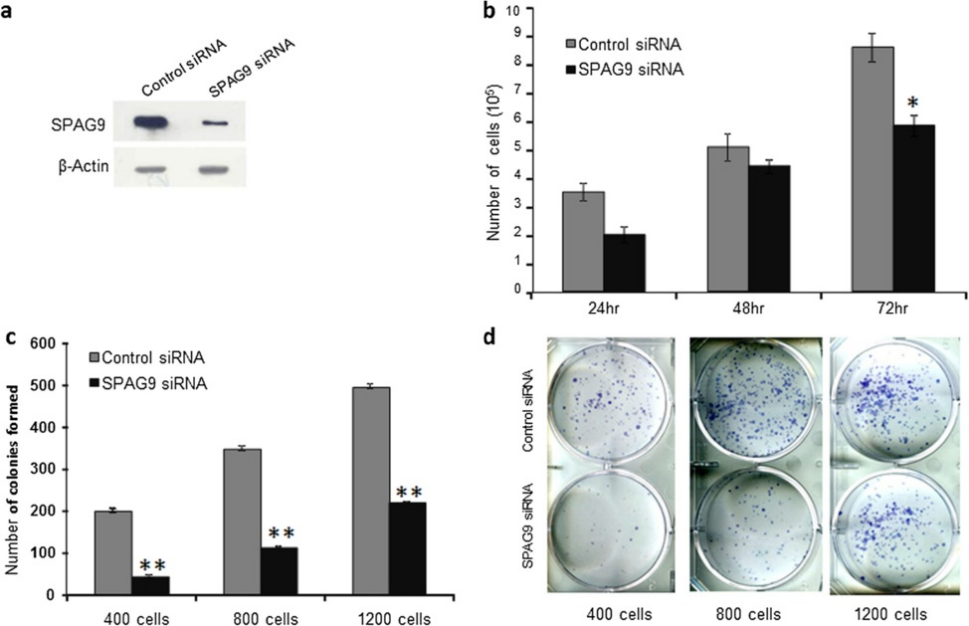
**Gene silencing of *SPAG9 *using plasmid-mediated siRNA approach.***SPAG9* specific siRNA (SPAG9 siRNA) and control siRNA (scrambled SPAG9) were used to transfect MDA-MB-231 breast cancer cells **(a)** No reduction in SPAG9 protein was observed in cells transfected with control siRNA. However, cells transfected with SPAG9 siRNA revealed ablation of SPAG9 protein. β-Actin protein was used as a loading control. **(b)** Knockdown of *SPAG9* inhibits cellular growth of breast cancer cells. Histogram plot clearly shows significant growth reduction (*P <* 0.01) in cells transfected with SPAG9 siRNA as compared to cells transfected with control siRNA. Results are representative of three independent experiments performed in triplicates. **(c)***SPAG9* knockdown reduces colony forming ability of breast cancer cells. MDA-MB-231 cells transfected with SPAG9 siRNA showed significantly reduced (*P <* 0.001) colony forming ability in 400–1200 cells in each histogram as compared to cells transfected with control siRNA. **(d)** A representative photomicrograph of colony forming ability treated with control siRNA or SPAG9 siRNA in 400, 800 and 1200 MDA-MB-213 cancer cells. Columns indicate mean (n = 3); bars, standard error. *; p < 0.01, **; p < 0.001 statistically significant compared with control siRNA. These results are representative of three independent experiments performed in triplicates.

Small interfering RNA mediated gene silencing approach was used to selectively knockdown *SPAG9* to study its role in cellular proliferation and colony forming ability. Highly aggressive triple-negative basal subtype MDA-MB-231 cells were used for *in vitro* gene silencing studies. SPAG9 siRNA construct transfected in MDA-MB-231 cells revealed ablation of SPAG9 protein as compared to control siRNA transfected cells as detected in Western blot analysis (Figure 2a). However, residual SPAG9 protein expression was also detected in SPAG9 siRNA transfected cells. Subsequently, MDA-MB-231 cells transfected with SPAG9 siRNA revealed significant reduction in cellular growth (*P* < 0.01) as compared to control siRNA transfected cells. Cell growth was reduced by 32% post 72 h of treatment (Figure 2b). Interestingly, colony forming ability was also significantly reduced by (*P* < 0.001) for various cell numbers seeded for MDA-MB-231 cells (59%-78% for 400–1200 cells) transfected with SPAG9 siRNA but not in cells transfected with control siRNA (Figure 2c; 2d). These results indicated that siRNA based knockdown of *SPAG9* resulted in significant reduction in cellular growth and colony forming ability of triple-negative MDA-MB-231 cells.

### Knockdown of *SPAG9* inhibits migration and invasion abilities of MDA-MB-231 cells

**Figure 3:**
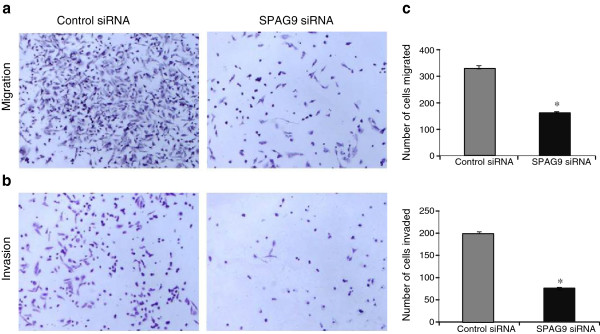
***SPAG9 *gene silencing significantly inhibited migration and invasion ability of MDA-MB-231 cells. (a)** Representative photomicrograph showed reduced number of migrated cells transfected with SPAG9 siRNA as compared to control siRNA transfected cells. A histogram shows significant reduction (*P* < 0.005) in the number of migrated cells transfected with SPAG9 siRNA as compared to control siRNA transfected cells. Observations based on three experimental triplicates. **(b)** Knockdown of *SPAG9* gene significantly reduced invasion of MDA-MB-231 cells through the Matrigel. Representative photomicrograph showed SPAG9 siRNA transfected cells exhibit reduced invasion abilities through Matrigel-coated Transwell filters as compared to control siRNA transfected cells. **(c)** A histogram shows significant reduction (*P <* 0.005) in the number of invaded cells in SPAG9 siRNA transfected MDA-MB-231 cells as compared to control siRNA transfected cells. Columns indicate mean (n = 3); bars, standard error. *; *P <* 0.005 statistically significant compared with control siRNA. These results are representative of three independent experiments performed in triplicates.

SPAG9 association with migratory and invasive abilities of MDA-MB-231 cells was further investigated. Our results showed a significant inhibition of 52.5% in migrating ability of MDA-MB-231 cells transfected with SPAG9 siRNA (*P* < 0.005) as compared to control siRNA as depicted in histogram (Figure 3a; 3c). Invasive ability of MDA-MB-231 cells was investigated using a reconstituted basement membrane barrier (Matrigel). Our results revealed a significant reduction of invasive ability (62.5%; *P* < 0.005) with SPAG9 siRNA as compared to control siRNA distinctly shown in histogram (Figure 3b; 3c). Our gene silencing studies collectively suggests that SPAG9 may be involved in migration and invasion of MDA-MB-231 cells.

### Gene silencing of *SPAG9* significantly reduces cellular motility

**Figure 4:**
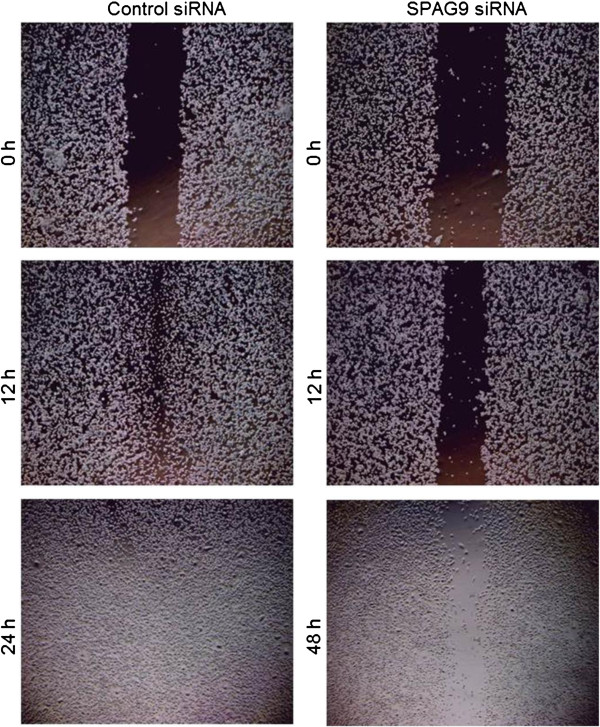
**Down regulation of *SPAG9 *causes reduction in wound healing capacity of MDA-MB-231 cells.** MDA-MB-231 cells transfected with SPAG9 siRNA showed significantly reduced cellular motility even after 48 h. In contrast, MDA-MB-231 cells transfected with control siRNA revealed closing of wound within 12 h. Results are from three independent experiments.

The important feature of metastasis process is the spread of tumor cells from the primary site to distant organs by cellular motility process. In order to investigate the role of SPAG9 in cellular motility, an *in vitro* wound healing assay was carried out. The motility of MDA-MB-231 cells was found to be significantly retarded when transfected with SPAG9 siRNA as compared to cells transfected with control siRNA. Our results revealed a closure of wound within 12h in control siRNA transfected cells, while MDA-MB-231 cells transfected with SPAG9 siRNA failed to close the wound scratch even after 48 h (Figure 4). This data clearly indicated that SPAG9 is involved in cellular motility and early spread of breast cancer cells, suggesting that SPAG9 may be involved in migration and invasion of MDA-MB-231 cells.

### SPAG9 depletion reduced tumor growth *in vivo*

**Figure 5:**
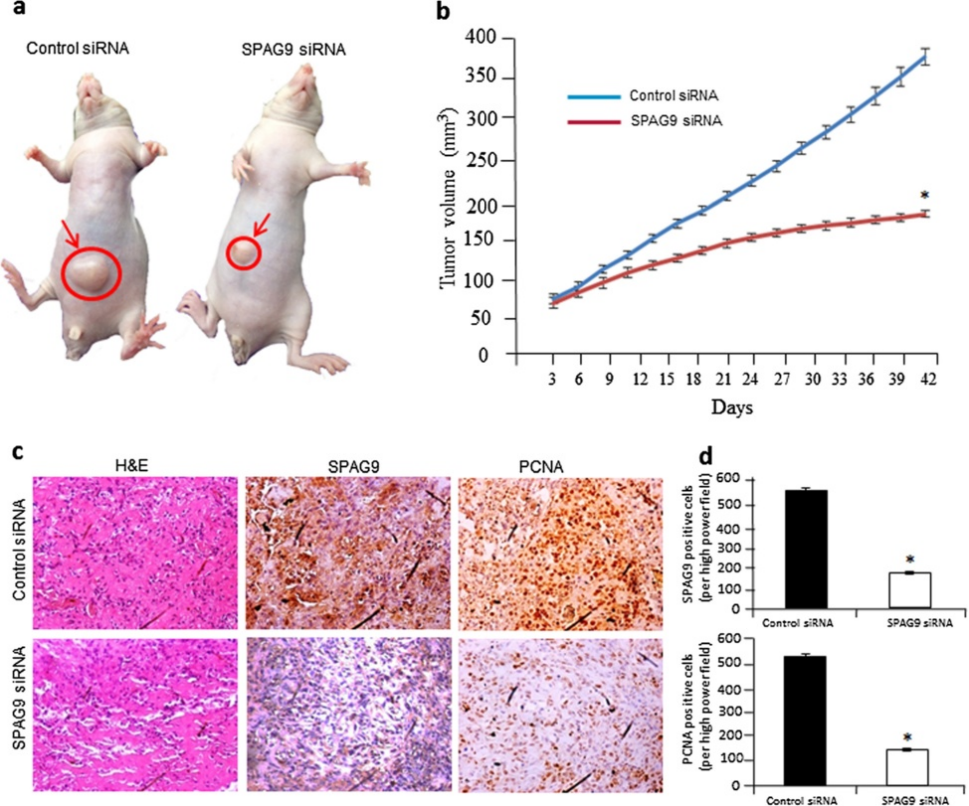
**Effect of down regulation of SPAG9 expression in breast cancer xenograft model. (a)** A representative photomicrograph showing nude mice with tumor (arrows) treated with control siRNA or SPAG9 siRNA plasmid. **(b)** a graph representing tumor volume calculated on the indicated days revealed significant reduction in the tumor growth in mice treated with SPAG9 siRNA plasmid compared with control siRNA (n = 8; *, *P* < 0.0001). **(c)** Immunohistochemical analysis of proliferating cell nuclear antigen (PCNA) and SPAG9 protein in control siRNA and SPAG9 siRNA treated tumors. Left panel- hematoxylin and eosin staining showing the histological cytostructure of the tumor of the control siRNA and SPAG9 siRNA treated mice. Middle panel- shows the reduced SPAG9 expression probed with anti SPAG9 antibody in SPAG9 siRNA treated mice tumors compared with control siRNA treated mice. Right panel- similarly shows the reduced PCNA expression in the SPAG9 siRNA treated mice compared with control siRNA treated mice. **(d)** The histograph representing the number of SPAG9 expressing cells and PCNA expressing cells. The histograph distinctly revealed the significantly reduced number of SPAG9 and PCNA expressing cells in SPAG9 siRNA compared with control siRNA treated mice. Columns indicate mean; bars, standard error. * *P* < 0.0001, statistical significant. Original magnification, × 400; objective × 40.

Our *in vitro* data indicated that ablation of SPAG9 expression by SPAG9 siRNA significantly reduced colony formation which led us to investigate its effect on human breast xenograft tumor growth in nude mice *in vivo*. To determine the effect of SPAG9 siRNA or control siRNA on tumor growth, mice were treated with control siRNA or SPAG9 siRNA and were observed for 42 days. A representative photograph shows reduced tumor growth in SPAG9 siRNA treated group compared with control siRNA treated group (Figure 5a). The tumor volume of mice injected with SPAG9 siRNA showed a significant reduction in tumor growth as compared to mice administered with control siRNA (Figure 5b; *P* < 0.001). Furthermore, in order to investigate whether the reduction of tumor growth is a result of ablation of SPAG9 expression, the xenograft tumors were excised and processed for immunohistochemical staining for SPAG9 protein expression. As depicted in Figure 5c, the SPAG9 protein was ablated in SPAG9 siRNA treated mice compared with mice treated with control siRNA. Furthermore to investigate whether SPAG9 siRNA treated animals which showed reduced tumor growth was associated with reduced cellular proliferation, serial tumor sections were probed for PCNA expression. Our data revealed that there was significant reduction of PCNA expression (72%; *P* < 0.0001) in tumors treated with SPAG9 siRNA treated compared with control siRNA as shown in Figure 5c and histograms (Figure 5d). These results suggest that SPAG9 may be a molecular target for novel cancer treatment modalities.

## Discussion

Breast cancer remains the major cause of death in women worldwide. Recent reports indicate that majority of cancer related deaths occur in economically weak and developing countries, such as India [1]. The existing treatment modalities for breast cancer patients are based on expression of ER, PR and HER2 molecules. However, a major challenge remains with the breast cancer patients with triple-negative tumors for which there are no or limited therapy available and have poor prognosis [15]. Therefore, in this regard we investigated the involvement of a well characterized CT antigen, SPAG9 in breast cancer using various breast cancer cell line models. Gene silencing approach was employed to study the association of SPAG9 with early spread and metastasis in highly aggressive triple-negative MDA-MB-231 breast cancer cells which may lead to new therapeutic strategies.

In our recent studies SPAG9 expression was shown to be associated with different stages and grades of various tumors [9]. In addition, SPAG9 was also shown to be associated with cellular proliferation, migration and invasion in squamous cell carcinoma-derived cervical cancer (SiHa) [12], renal cell carcinoma (Caki-1) [11] and colon cancer (COLO 205 and HCT 116) [13] cell line models, respectively. Recently, we also demonstrated an association of SPAG9 with early spread of breast carcinogenesis [14]. Collectively, our data indicates that SPAG9 may be a potential key molecule contributing towards the early spread and metastasis. In this context, we investigated SPAG9 expression in breast cancer cells of different histological subtypes, harboring different hormone receptor.

So far very few studies have proposed an association of CT antigens with cellular growth, migration and invasion abilities in various breast cancer cell lines. Earlier, *X antigen family, member 1* (*XAGE-1*) was shown to be expressed only in ER-negative breast cancer cell lines (MDA-MB-231, SK-BR-3, and MDA-MB-468 cells), and no expression in ER-positive breast cancer cell lines (ZR-75-1, MCF-7, and BT-474 cells) [16] suggesting that *XAGE-1* transcription may be functional through estrogen receptor pathway. Yet another well studied CT antigens, melanoma antigen family A (MAGEA) and New York oesophageal squamous cell carcinoma 1 (NY-ESO-1) were reported to have expression in ER- negative breast cancer cells [BT20 cells [17]. In contrast, it is important to mention that in our study, SPAG9 expression was detected in all breast cancer cells, independent of their hormone receptor status or HER2 expression pattern. Our RT-PCR results confirmed *SPAG9* mRNA expression in all breast cancer cells which was further validated for protein expression by Western blotting and IIF. We did not find any discrepancy between SPAG9 mRNA and protein expression in all breast cancer cells. Further, our FACS data revealed that SPAG9 protein was also localized on the plasma membrane of MCF-7, MDA-MB-231, BT-474 and SK-BR-3 breast cancer cells, indicating its putative use in development of immunotherapeutic target for breast cancer treatment.

Metastasis is a complex process involving multiple steps including epithelial mesenchymal transition (EMT) and mesenchymal epithelial transition (MET) resulting in migration, invasion, colony forming abilities and subsequently tumor growth at distant sites [18]. In this context, it is important to investigate gene and gene products involved in early spread, tumor progression and metastasis. Plasmid-based siRNA approach was used to selectively knockdown the expression of *SPAG9* in MDA-MB-231 cells. Gene silencing approach has been employed in few studies to investigate the biological role of CT antigens in tumorigenesis and their effects on tumor progression. In a recent study, knockdown of *MAGE-D4B* in triple-negative breast cancer cell line model Hs578T demonstrated a significant reduction in colony forming and invasive abilities [19]. Further, employing gene silencing approach, the role of well characterized CT antigens, MAGE-C1 and MAGE-A3 were shown to promote cellular growth and colony forming ability in myeloma cells (Molp-8 and KMS-12-BM cells) [20]. Knockdown of *synovial sarcoma X* (*SSX*) in melanoma cells (DFW) also showed reduction in cell migration [21]. Similarly, significant reduction in cellular motility by wound healing assay was demonstrated by knockdown of *sperm-associated antigen 1* (*SPAG1*) suggesting a strong association of SPAG1 with migration abilities in pancreatic cancer cells, Panc1 [22]. It is important to mention that none of the earlier studies demonstrated the effect of knockdown of CT antigen on all of the key features of metastasis except a recent study [23] suggesting the role of Melanoma antigen gene-A3 (MAGE-A3) gene in invasion and angiogenesis. Similarly, our study also revealed the involvement of SPAG9 in cellular proliferation and migration suggesting its potential role in early spread. Interestingly our study showed that SPAG9 is involved in invasive potential of MDA-MB-231 cells and down regulation of *SPAG9* significantly reduced the cellular growth, colony forming ability, migratory and invasive ability and wound healing capacity in these cells. Furthermore, knockdown of SPAG9 expression *in vivo* in nude mice also showed reduction in tumor growth indicating its important role in breast cancer.

In conclusion, to the best of our knowledge, this is the first report where we have put forth an evidence of potential role of SPAG9 in cellular growth, migration, invasion and colony forming ability in highly aggressive triple-negative MDA-MB-231 breast cancer cells. Furthermore we also demonstrated that SPAG9 expression was higher in all breast cancer cell compared to normal mammary epithelial cells. In addition, *in vivo* xenograft studies further strengthen the role of SPAG9 in breast cancer. Our study provides an association between SPAG9 expression and its potential role in breast cancer, and thus lays a foundation for developing a promising therapeutic target for triple-negative breast cancer.

## Competing interests

The authors declare that they have no competing interests.

## Authors’ contributions

AS performed SPAG9 expression studies and drafted the manuscript. SA carried out transfection studies and in vitro and in vivo experiments. AV performed RT-PCR analysis. DP performed real time PCR. SS and NJ analyzed the data and prepared the figures. AKA and NKL helped in drafting the manuscript. AS conceived the study, designed the experiments and prepared the manuscript. All authors read and approved the final manuscript.
